# Noncommunicable Disease Emergencies During Arbaeenia Mass Gathering at Public Hospitals in Karbala, Najaf, and Babel Governorates, Iraq, 2014: Cross-Sectional Study

**DOI:** 10.2196/10890

**Published:** 2019-09-30

**Authors:** Faris Lami, Abdul Wahhab Jewad, Abulameer Hassan, Hadeel Kadhim, Sura Alharis

**Affiliations:** 1 Department of Community and Family Medicine College of Medicine University of Baghdad Baghdad, Iraq; 2 Najaf Directorate of Health Iraq Ministry of Health Najaf, Iraq

**Keywords:** mass gathering, Iraq, noncommunicable diseases

## Abstract

**Background:**

Arbaeenia is the largest religious mass gathering (MG) in Iraq where millions of people from Iraq and many other countries visit Karbala city, south Iraq. MGs are associated with high rates of morbidity and mortality from different noncommunicable diseases (NCDs) such as cardiovascular diseases, diabetes mellitus, and asthma. There is a scarcity of publications that address MGs in Iraq.

**Objective:**

This study aimed to explore the NCD emergencies in public hospitals in Karbala, Najaf, and Babel governorates in Iraq, during the Arbaeenia MG and assess predisposing factors for NCD emergencies.

**Methods:**

The study was conducted from November 27 to December 16, 2014. Data were collected in the pre-event and during MG event from 7 selected hospitals. The pre-event data were collected from emergency room (ER) registers and logbooks, and the data on the MG event were collected daily through direct interview with patients and treating physicians using a structured questionnaire.

**Results:**

In total, 4425 NCD emergencies were recorded. Of these, 80.13% (3546/4425) were collected during the MG event. The NCD emergencies attended at ER hospitals during MG were severe hypertension (HT), diabetes (hyperglycemia), ischemic heart disease (IHD), asthma, and pulmonary edema. The load of NCD emergencies and the daily average emergencies increased 4-fold and 2-fold during the MG event, respectively. Most of the NCD emergencies were treated at ER departments, and a few were hospitalized. Intense physical activities and poor adherence to diet and medication were risk factors for IHD, severe HT, and hyperglycemic diabetes emergencies. Exposure to noxious gases or fumes and recent respiratory infections were risk factors for asthma emergencies.

**Conclusions:**

As the pilgrims approached Karbala city during the Arbaeenia MG, the hospitals on the roads leading to the city experienced an increased load of patients because of different NCD emergencies. Although hospitals should be equipped with the necessary supplies, health education for pilgrims is mandatory, particularly on the factors that can exacerbate their diseases.

## Introduction

### Background

The World Health Organization defines mass gatherings (MGs) as “events attended by a sufficient number of people to strain the planning and response resources of a community, state or nation” [1]. MGs carry potential health risks to the local population and to the global community [2-4]. Such gatherings pose a significant public health threat to the host country and the attendees’ countries of origin [2,3,5-8]. The nature of MGs can be political rallies, social events, religious activities, sporting events, and others [5]. Organizers of most MGs, especially those of a longer duration such as the Hajj, Olympics, or the Kumbh Melas in India, offer basic health services to the attendees [9,10]. Individuals attending the MGs and the host population are at risk of communicable disease infections, injuries, and exacerbations of noncommunicable diseases (NCDs). MGs have been associated with high rates of morbidity and mortality from NCDs, accidents, and terrorist attacks [11].

During the Hajj MG, high rates of morbidity and mortality from NCDs, such as cardiovascular diseases, diabetes mellitus (DM), and asthma were reported [2,3,12-14,15-17]. Attendees traveling to the event from outside the country may be exposed to a different environment in addition to altering their daily routine and diets [18,19]. Walking long distances, changes in daily activities, and poor adherence to diet and medications could negatively impact NCD control, particularly hypertension (HT), DM, and ischemic heart diseases (IHDs) [18]. The incidence of severe acute cardiovascular events is more than doubled during MGs for people exposed to intense stress [2,20,21].

In Iraq, religious MGs occur throughout the year, mainly in Karbala, Najaf, and Baghdad. Arbaeenia is the largest religious MG and convenes in Karbala annually [5]. During Arbaeenia, Shi’a Muslims commemorate the 40 days following the day of the martyrdom of Hussein bin Ali, the grandson of the Prophet Muhammad, at the Battle of Karbala in 61 AH (680 CE) [5].

More than 10 million people, mainly from Iraq, visit Imam Hussein’s shrine in Karbala. Attendees walk hundreds of kilometers for many days to reach Karbala. These visitors are exposed to risks that could affect their health including intense physical activity and poor adherence to diet regimes and medications. The Arbaeenia MG follows the Islamic lunar calendar, and the date moves forward by 10 to 11 days every year, therefore presenting health risks associated with seasonal variation [5,22].

### Objectives

The public health impact of religious MGs in Iraq has rarely been studied [5]. This study aimed to describe NCD emergencies during the Arbaeenia MG and to assess predisposing factors for NCD emergencies.

## Methods

### Setting and Data Collection

The study was conducted from November 27 to December 16, 2014, in 3 Iraqi governorates: Karbala, Najaf, and Babel, located to the south of Baghdad, with a population of over a million each. We implemented the study in two phases, the pre-event phase that covered the first 7 days of the survey and the event phase covering 13 days, during their journey to the event. The pre-event phase serves as a baseline for NCD emergencies and admissions; the event phase captures the burden of NCDs during the MG.

Data on all patients attended for NCD diagnoses at emergency rooms (ERs) or admitted to 1 of the 7 hospitals selected for the study were collected. The selected hospitals included the 4 main Ministry of Health (MOH) hospitals located at the center of selected governorates and 3 small hospitals located along the road to Karbala. A total of 3 of the selected hospitals (Al-Seder, Al-Hakeem, and Al-Haideria) were located in Najaf, 3 in Babel (Hashmia, Al-Qasim, and Merjan), and 1 in Karbala (Al-Hussein Hospital). 

NCD emergencies include the following 6 groups: HT or hypotension; DM: hypoglycemia, ketoacidosis, and hyperosmolar syndrome; IHD: angina pectoris, myocardial infarction (MI), arrhythmias; cerebrovascular accident (CVA): ischemic and hemorrhagic accidents; chronic obstructive pulmonary disease (COPD): asthma and other intermittent, persistent COPDs; and cardiogenic pulmonary edema (PE): heart failure owing to IHDs.

Data on the pre-event phase were collected by a team of 6 Field Epidemiology Training Program graduates and current officers led by the principal investigator. Data were abstracted from available registers and logbooks of the 7 selected hospitals in the pre-event phase. During the event phase, data were collected daily (24 hours) by direct interview with patients or accompanying relatives and treating physicians using a questionnaire, which was filled by trained health personnel.

### Data Variables

The information collected included age, sex, residence, provisional diagnosis obtained from treating physicians, and potential predisposing factors that might have led to development of the NCD emergency, which included intense physical activity, poor adherence to dietary regimen, poor adherence to medications, exposure to noxious gases or fumes, and history of recent respiratory infection (RI).

### Statistical Analysis

Epi Info developed by US Center of Diseases Control and Prevention, Atlanta, Georgia and SPSS acquired by IBM were used for data entry and analysis. We estimated the daily average number of patients with NCDs attending the ER by study phase and tested the differences between the daily average patients in the pre-event and event study phase by demographics and by hospitals or departments. We used a *t* test to test statistical differences at *P*<.05. We estimated the risk ratio (RR) and 95% CIs of predisposing factors for NCD emergencies.

Official approval was obtained from the Iraqi MOH before implementation of the study. Confidentiality of data throughout the study was assured, and access to data was limited to research team members.

## Results

Tables 1 and 2 show the number and daily average for NCD emergencies by demographics and hospitals and by study phase. There were 4425 NCD patients reported in the study period, of which 80.13% (3546/4425) were reported during the event phase, a 4-fold increase. The hospitals attended 211 patients on average per day. The daily average number of patients was more than 2-fold during the event phase compared with the pre-event, 273 and 126 cases, respectively, *P*<.001. The significant differences in the daily average number of NCD emergencies between the pre-event and event phase remained significant across age groups, gender, and residence (*P*<.001).

The majority of patients (76.43%, 3382/4425) were aged 40 years and older and 5.11% (226/4425) were younger than 20 years. The male and female ratio was 1.2:1. A larger number of males were observed during the event phase, whereas a similar ratio of both sexes was observed in the pre-event phase. Almost three-quarter of the cases was residents of the 3 governorates: Najaf, Babel, and Karbala. About 10.32% (457/4425) were foreigners.

Karbala attended the highest number of NCD emergencies (37.45%, 1657/4425) reported by the single participating hospital in the study, and Babel reported the least (26.31%, 1164/4425). The daily average of NCD emergencies between the pre-event and event differed significantly in Najaf (*P*=.006), Karbala (*P*<.001), and Babel (*P*=.03). The largest daily average number of NCD emergencies were attended in Al-Hussein hospital in Karbala, 83 cases, whereas Al-Qasem hospital attended the least, 9 cases. Both Al-Hussein and Al-Qasem hospitals attended 3.44% (1657/4425) and 3.93% (174/4425) of the total cases, respectively (Table 2). The daily average number of NCD emergencies between the pre-event and event study phases differ significantly in Al-Hussein (*P*<.001) and Al-Haideria hospitals (*P*=.008).

The majority (91.71%, 4058/4425) of NCD emergencies were attended at the ER, and 8.29% (367/4425) were hospitalized. Daily admissions to hospitals did not vary by study phase and remained at around 18 patients per day. In contrast, the average daily cases attended at the ERs increased from 107 patients in the pre-event to 254 patients in the event phase (*P*<.001).

**Table 1:** Distribution of number and daily average of noncommunicable disease emergencies by demographics and study phase, Iraq 2014.

Demographics		Pre-event		Event		Total		*P* value^a^
		n (%)	Daily average	n (%)	Daily average	n (%)	Daily average	
**Age group (years)**								
	<20	54 (6.1)	8	172 (4.85)	13	226 (5.11)	11	.02
	20-39	162 (18.4)	23	655 (18.47)	50	817 (18.46)	41	<.001
	40-49	339 (38.6)	48	1411 (39.79)	109	1750 (39.55)	88	<.001
	≥60	324 (36.9)	46	1308 (36.89)	101	1632 (36.88)	82	<.001
**Sex**								
	Male	443 (50.4)	63	1960 (55.27)	151	2403 (54.31)	120	<.001
	Female	436 (49.6)	62	1586 (44.73)	122	2022 (45.69)	101	<.001
**Residence**								
	Karbala-Najaf-Babel	794 (90.3)	113	2437 (68.73)	187	3231 (73.02)	162	<.001
	Other Iraq Governorates	59 (6.7)	8	678 (19.12)	52	737 (16.65)	37	<.001
	Foreign countries	26 (2.9)	4	431 (12.15)	33	457 (10.33)	23	<.001
**Reporting governorates**								
	Najaf	358 (40.7)	51	1246 (35.14)	96	1604 (36.25)	80	.006
	Karbala	198 (22.5)	28	1459 (41.14)	112	1657 (37.45)	83	<.001
	Babel	323 (36.8)	46	841 (23.72)	65	1164 (26.30)	58	.03
Total		879 (100.0)	126	3546 (100.00)	273	4425 (100.00)	221	<.001

^a^*P* value (*t* test) testing differences between daily average in the pre-event and event study phase.

**Table 2:** Distribution of number and daily average of noncommunicable disease emergencies by hospitals and study phase, Iraq 2014.

Hospitals		Pre-event		Event		Total		*P* value^a^
		n (%)	Daily average	n (%)	Daily average	n (%)	Daily average	
**Hospital**								
	Al Haideria	34 (3.9)	5	504 (14.21)	39	538 (12.15)	27	.008
	Al Hakeem	216 (24.6)	31	513 (14.46)	39	729 (16.47)	36	.42
	Al Hashmia	102 (11.6)	15	377 (10.63)	29	479 (10.82)	24	.06
	Al Hussien	198 (22.5)	28	1459 (41.14)	112	1657 (37.44)	83	<.001
	Al Qasim	47 (5.4)	7	127 (3.58)	10	174 (3.93)	9	.28
	Al Seder	108 (12.3)	15	229 (6.45)	18	337 (7.61)	17	.59
	Merjan	174 (19.8)	25	337 (9.50)	26	511 (11.54)	26	.85
	Total	879 (100.0)	126	3546 (100.00)	273	4425 (100.00)	221	<.001
**Hospital department**								
	Emergency room	751 (85.4)	107	3307 (93.26)	254	4058 (91.70)	203	<.001
	Admissions	128 (14.6)	18	239 (6.73)	18	367 (8.29)	18	.98
	Total	879 (100.0)	126	3546 (100.00)	273	4425 (100.00)	221	<.001

^a^*P* value (*t* test) testing differences between daily average in the pre-event and event study phase.

Table 3 shows the percent distribution and the daily average of types of NCD emergencies during the pre-event and event phases. Among the reported NCD emergencies, blood pressure–related emergencies (hypotension or HT) were the most frequently encountered (36.04%, 1595/4425), of which 29.04% (1285/4425) was severe HT and 7.00% (310/4425) low blood pressure. IHDs were the second highest reported NCD emergencies (21.10%, 934/4425), of which angina pectoris constituted 11.77% (521/4425), arrhythmias 5.49% (243/4425), and MI 3.84% (170/4425). Around 19.23% (851/4425) of NCD emergencies were asthma, and 16.43% (727/4425) were DM, of which hyperglycemia with and without ketoacidosis accounted for 1.78% (654/4425) of all reported emergencies, and hypoglycemic emergencies were less than 2% (1.64%, 73/4425) of all cases. PE and CVAs were the least reported emergencies, 4.00% (177/4425) and 3.16% (140/4425), respectively. The daily average number of patients in the pre-event and event phase varied significantly in IHDs, DM, hypotension or HT, asthma, and PE, *P*<.05.

Table 4 shows the number and percent distribution of predisposing factors to the NCD emergencies. Of the cases who had intense physical activity, 52.1% (321/616) experienced IHD, 4.9% (30/616) experienced severe HT, 0.8% (5/616) experienced hyperglycemia, 12.3% (76/616) experienced asthma, 3.1% (19/616) experienced PE, and 1.8% (11/616) experienced CVA. Of those who reported poor adherence to diet, 22.5% (211/936) had IHD, 36.5% (342/936) had severe HT, 27.9% (261/936) had hyperglycemia, 2.3% (22/936) had asthma, and PE and CVA had around 1% each. For those who reported poor adherence to medication, 17.07% (241/1412) had IHD, 44.05% (622/1412) had severe HT, 20.89% (295/1412) had hyperglycemia, 10.69% (151/1412) had asthma, 5.24% (74/1412) had PE, and 1.48% (21/1412) had CVA. Of those exposed to noxious gases, 94.9% (111/117) reported asthma and 5.1% (6/117) reported PE. In addition, of the cases who had a history of recent RI, 92.8% (271/292) reported asthma and 3.4% (10/292) reported PE.

Table 5 shows the risk ratio of predisposing factors for NCD emergencies. Patients who reported intense physical activity were more likely to have IHD emergencies (RR=3.1, 95% CI 2.8-3.5) and less likely to have severe HT (RR=0.14, 95% CI 0.1-0.2), hyperglycemia (RR=0.04,95% CI, 0.02-0.1), and asthma emergencies (RR=0.6, 95% CI 0.5-0.8) compared with those who did not report intense physical activity. Patients who reported poor adherence to diet were more likely to have severe HT (RR=1.4, 95% CI 1.2-1.5) and hyperglycemia (RR=2.3, 95% CI 1.9-2.6) and less likely to have asthma (RR=0.1, 95% CI 0.06-0.1) and PE emergencies (RR=0.1, 95% CI 0.05-0.3) compared with patients who did not report poor adherence to diet. Patients with poor adherence to medications were more likely to have higher severe HT (RR=2.3, 95% CI 2.1-2.6), hyperglicemia (RR=1.5, 95% CI 1.3-1.8), asthma (RR=1.4, 95% CI 1.2-1.7), and PE emergencies (RR=2.6, 95% CI 1.8-3.8) and less likely to have IHD emergencies compared with those who did not report poor adherence to medication. Patients who had exposure to noxious gases had higher risk for asthma (RR=5.9, 95% CI 5.4-6.5) compared with those who did not report exposure. Similarly, patients who had a history of recent RI had higher risk for asthma (RR=7.9, 95% CI 7.2-8.8) compared with those who did not report a history of recent RI.

**Table 3:** Distribution of number and daily average of noncommunicable disease emergencies by study phase.

NCD^a^ emergencies		Pre-event		Event		Total		*P* value^b^
		n (%)	Daily average	n (%)	Daily average	n (%)	Daily average	
**Ischemic heart disease**		199 (22.7)	28	735 (20.72)	57	934 (21.11)	47	<.001
	Angina	104 (11.9)	15	417 (11.75)	32	521 (11.77)	26	.006
	Arrhythmias	61 (6.9)	9	182 (5.13)	14	243 (5.49)	12	.07
	Myocardial infarction	34 (3.9)	5	136 (3.83)	10	170 (3.84)	9	<.001
**Hypotension/ hypertension**		296 (33.7)	42	1299 (36.63)	100	1595 (36.05)	80	<.001
	Severe hypertension	223 (25.4)	32	1062 (29.94)	82	1285 (29.04)	64	<.001
	Hypotension	73 (8.3)	10	237 (6.68)	18	310 (7.00)	16	.03
**Diabetes mellitus**		124 (14.1)	18	603 (17.00)	46	727 (16.43)	36	<.001
	Hyperglycemia	112 (12.8)	16	542 (15.28)	42	654 (14.78)	33	<.001
	Hypoglycemia	12 (1.4)	2	61 (1.72)	5	73 (1.65)	4	.01
Asthma		166 (18.9)	24	685 (19.31)	53	851 (19.23)	43	<.001
Pulmonary edema		40 (4.6)	6	137 (3.86)	11	177 (4.00)	9	.02
Cerebrovascular accidents		53 (6.0)	8	87 (2.45)	7	140 (3.16)	7	.60
Total		878 (100.0)	125	3546 (100.00)	273	4424 (100.00)	221	<.001

^a^NCD: noncommunicable disease.

^b^*P* value is based on the *t* test.

**Table 4:** Number of noncommunicable disease emergencies by predisposing factors.

Noncommunicable disease emergency		Potential predisposing factors, n (%)					
		Intense physical activity	Poor adherence to diet	Poor adherence medications	Exposure to noxious gases	History of exposure to recent respiratory infection	Total responses
**Ischemic heart disease**		321 (52.1)	211 (22.5)	241 (17.06)	0 (0.0)	8 (2.7)	781 (23.15)
	Angina	173 (28.0)	124 (13.2)	138 (9.77)	0 (0.0)	6 (2.0)	441 (13.07)
	Arrhythmias	81 (13.1)	54 (5.7)	48 (3.39)	0 (0.0)	0 (0.0)	183 (5.42)
	Myocardial infarction	67 (10.8)	33 (3.5)	55 (3.85)	0 (0.0)	2 (0.6)	157 (4.65)
**Hypotension/hypertension**		151 (24.5)	407 (43.4)	628 (44.47)	0 (0.0)	2 (0.6)	1188 (35.22)
	Severe Hypertension	30 (4.8)	342 (36.5)	622 (44.05)	0 (0.0)	1 (0.3)	995 (29.49)
	Hypotension	121 (19.6)	65 (6.9)	6 (0.42)	0 (0.0)	1 (0.3)	193 (5.72)
**Diabetes mellitus**		38 (6.1)	278 (29.7)	297 (21.03)	0 (0.0)	1 (0.3)	614 (18.20)
	Hyperglycemia	5 (0.8)	261 (27.8)	295 (20.89)	0 (0.0)	1 (0.3)	562 (16.66)
	Hypoglycemia	33 (5.3)	17 (1.8)	2 (0.14)	0 (0.0)	0 (0.0)	52 (1.54)
Asthma		76 (12.3)	22 (2.3)	151 (10.69)	111 (94.8)	271 (92.8)	631 (18.70)
Pulmoary edema		19 (3.0)	5 (0.5)	74 (5.24)	6 (5.1)	10 (3.4)	114 (3.37)
Cerebrovascular accidents		11 (1.7)	13 (1.3)	21 (1.48)	0 (0.0)	0 (0.0)	45 (1.33)
Total responses		616 (100.0)	936 (100.0)	1412 (100.00)	117 (100.0)	292 (100.0)	3373 (100.00)

**Table 5:** Risk ratio of predisposing factors for noncommunicable disease emergencies.

Potential risk factors	Risk ratio (95% CI)					
	Ischemic heart diseases	Severe hypertension	Diabetes mellitus (hyperglicemia)	Asthma	Pulmonary edema	Cerebrovascular accidents
Intense physical activity	3.1 (2.8-3.5)	0.14 (0.1-0.2)	0.04 (0.02-0.1)	0.6 (0.5-0.8)	0.9 (0.6-1.6)	1.4 (0.7-2.8)
Poor adherence to diet	1.0 (0.8-1.1)	1.4 (1.2-1.5)	2.3 (1.9-2.6)	0.1 (0.06-0.1)	0.1 (0.05-0.3)	1.1 (0.6-2.2)
Poor adherence to medications	0.6 (0.5-0.7)	2.3 (2.1-2.6)	1.5 (1.3-1.8)	1.4 (1.2-1.7)	2.6 (1.8-3.8)	1.2 (0.7-2.2)
Exposure to noxious gases	—^a^	—	—	5.9 (5.4-6.5)	1.5 (0.7-3.4)	—
History of exposure to recent respiratory infection	—	—	—	7.9 (7.2-8.8)	1.0 (0.5-1.9)	—

^a^Not applicable.

## Discussion

### Principal Findings

This is the first study to describe NCD emergencies during MG events in Iraq. The most common emergencies attended in the hospitals during the event were HT, IHD, asthma, and diabetes (hyperglycemia). The load of the NCD emergencies during the MG event increased 4-fold, and the daily average cases also doubled compared with the pre-event. This indicates the immense burden of NCD emergencies to hospitals during MG events and the need for adequate planning of services by the government of Iraq. This is beyond the MOH capacity and domain and needs the participation of other ministries and governmental agencies.

The daily average for NCD emergencies increased 2-fold in IHD and asthma and almost 3-fold in severe HT and diabetes (hyperglycemia) during the event. These findings are consistent with other studies conducted in the region during religious MG events [5,13,14,23]. Severe HT and diabetes (hyperglycemia) are likely to be sensitive to changes in physical activity, diet and medications, which are disrupted during the MG.

The study shows that intensive physical activity leads to IHD emergency but is inversely correlated with severe HT and diabetic-hyperglycemia emergencies during the MG event. Regular exercise controls HT and diabetic hyperglycemia and improves IHD, but sudden physical activities in individuals with cardiovascular diseases might precipitate heart attack [17]. Poor adherence to diet and medications for hypertensive, diabetics, and asthmatic patients predispose to ER attendance for treatment in the MG event. Similarly, exposure to noxious gases or fumes, which is obvious in Iraq MG, and history of recent RI lead to ER attendance for asthma treatment. Individuals with NCDs should be accompanied by caring relatives, in addition to receiving regular checkups at mobile clinics to avoid medical emergencies and premature deaths.

The risk for medical emergencies and hospital admissions during MG are often related to the size of the masses, climatic conditions, demographics, services availability, prior planning, and behaviors of the attendees. The purpose of the MG studied was religious, where masses march orderly, but the lack of prior planning for service needs, harsh climatic conditions, long distance walk, lack of adequate services, and overcrowding may have contributed to medical emergencies observed in the study. Setting up temporary resting places, mobile clinics, and maintaining sufficient supply of health care staff could minimize the need for ER medical services and hospitalizations, which could be addressed through proper planning and implementation.

Most of the NCD emergencies were handled in the ER departments and few were admitted to the hospitals, which is similar to a study conducted in Iraq during the Ashuraa religious MG [5]. The majority of the NCD cases were aged younger than 50 years and more likely to be healthy, which may have contributed to low hospitalizations.

### Limitations

The study has some limitations. The increased load during the event phase was solely related to patients with NCD emergencies attending ER departments. Some NCD emergency cases may have been missed because of the chaotic conditions at the ER departments during the MG. Some patients attended in ER may have also been admitted to the hospitals after the data were collected, affecting the percent of admissions. As this study was confined to the hospitals, NCD emergency cases treated in mobile clinics were not counted; thus, the increase of cases in the event could be higher than the numbers reported. The study was limited to 7 hospitals, and the load of NCD emergencies attended in other hospitals may result in different estimates of the effect of MG on NCD emergencies.

### Conclusions

In conclusion, with the pilgrims approaching their destination to Karbala city during Arbaeenia MG, the hospitals on the roads leading to the city experienced an increased load of patients because of different NCD emergencies. Although the MOH is more concerned with outbreaks of communicable diseases during MGs, NCDs should be seriously considered in the MGs planning. The hospitals should be fully equipped with the necessary supplies and trained health workers. Furthermore, health education for pilgrims is mandatory, particularly on the factors that can exacerbate their diseases.

## Abbreviations

COPD: chronic obstructive pulmonary disease
CVA: cerebrovascular accident
DM: diabetes mellitus
ER: emergency room
HT: hypertension
IHD: ischemic heart disease
MG: mass gathering
MI: myocardial infarction
MOH: Ministry of Health
NCD: noncommunicable disease
PE: pulmonary edema
RI: respiratory infection
RR: risk ratio
